# Prognostic significance of poorly differentiated histology and impact of adjuvant chemotherapy in early squamous cell carcinoma of cervix uteri

**DOI:** 10.1002/cam4.3780

**Published:** 2021-03-18

**Authors:** Hui Luo, Hongxia Yao, Xinxin Xu, Zhen Li, Hongqin Zhao, Haiyan Zhu

**Affiliations:** ^1^ Department of Gynecology Shanghai First Maternity and Infant Hospital Tongji University School of Medicine Shanghai China; ^2^ Department of Gynecology the First Affiliated Hospital of Wenzhou Medical University Wenzhou China

**Keywords:** chemotherapy, low‐risk, poor differentiation, squamous cervical cancer

## Abstract

**Objective:**

This study is to determine whether the addition of cisplatin‐based chemotherapy after radical hysterectomy will improve the survival of low‐risk squamous cervical carcinoma with poor differentiation.

**Methods:**

Patients with low‐risk squamous cervical cancer (FIGO IA2–IIA, absent high‐ and intermediate‐risk factors after pathological evaluation) were eligible for this study. As first, the prognostic relevance of G3 versus G1/G2 among patients with low‐risk squamous cervical cancer was analyzed, then, the oncological results of postoperative chemotherapy among low‐risk squamous cervical cancer with poor differentiation was explored.

**Results:**

Totally, there were 367 low‐risk squamous cervical cancer patients, of whom 161 were poor‐differentiated (47 in the chemotherapy group and 114 in the nonchemotherapy group), with a median follow‐up time of 56 months. Patients with G3 displayed a significantly worse overall survival (*p* = 0.035), and a higher recurrence rate (*p* = 0.014) than patients with G1/G2. Compared with the nonchemotherapy group, the hazard ratios (95%CI) for recurrence‐free survival in the chemotherapy group was 0.24 (0.06–0.93), (*p* = 0.038). No difference in overall survival was observed between the chemotherapy group and the nonchemotherapy group.

**Conclusions:**

The addition of cisplatin‐based chemotherapy following surgery significantly improved recurrence‐free survival for low‐risk, poor differentiation, and early stage squamous cervical cancer patients.

## Graphical abstract

The addition of cisplatin‐based chemotherapy following surgery significantly improved recurrence‐free survival for low‐risk, poor differentiation, early‐stage squamous cervical cancer patients.

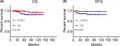

## 1 INTRODUCTION

Early stage of cervical carcinoma patients usually showed a relatively better prognosis, and the 5‐year survival rate is almost around to 85%.^1^ However, those with high‐risk factors, such as surgical margins, parametrial invasion, and lymph node metastases, increase the risk of recurrence to 50%–70%.^2^ Besides, patients with intermediate‐risk factors, including large diameter of tumor, deep invasion of stroma, as well as LVSI, increase the recurrence to approximately 30%.^3^, ^4^ Accordingly, for patients with intermediate or high risk of recurrence, adjuvant treatment is recommended. Nevertheless, there are few studies on low‐risk cervical cancer, which are defined as the absence of high‐risk factors and intermediate‐risk factors.

In early 1991, Smiley et al. identified a degree of differentiation as the only histopathologic factor correlated with recurrence among 95 patients with stage IB squamous cervical cancer with low recurrence rate.^5^ Recently, Matsuo et al. showed that the cause‐specific survival increased to 4.48 times among patients with poor differentiation as compared to those with well‐differentiation^6^ by analyzing 4,045 low‐risk squamous cervical cancer.^6^ These data raised the question that whether adjuvant therapy is needed in a low‐risk squamous cervical cancer patient with poor differentiation. Considering that nearly half of the patients had poorly tumor differentiation to avoid overtreatment, the application of tumor differentiation as a standard of adjuvant therapy in the low‐risk population was compromised.^5^ Therefore, few studies explored the adjuvant therapy in these patients.

Currently, chemotherapy has been widely used in cervical cancer patients as a supplement to definitive locoregional treatments (surgical operation or radiotherapy) to improve their effect,^7^ in which, cisplatin combined with paclitaxel was the most effective chemotherapy regimen for recurrent, or metastatic squamous cell carcinoma of the cervix to date. In the current study, we first assessed the prognostic relevance of G1/G2 versus G3 among low‐risk squamous cervical cancer patients, then, explored the oncological results of adjuvant chemotherapy after radical hysterectomy among low‐risk squamous cervical cancer with poor differentiation.

## 2 METHODS

### 2.1 Patients

We retrospectively reviewed the records of 1465 squamous cervical cancer patients with stage IA2–IIA (according to 2009 FIGO staging criteria) underwent radical hysterectomy and pelvic lymphadenectomy at the First Affiliated Hospital of Wenzhou Medical University from 2008 to 2018. All these patients were pathologically confirmed invasive cervical cancer. After pathological assessment, these patients were classified as following: high‐risk, intermediate‐risk, and low‐risk. According to Peters’ criteria, the criteria for high‐risk tumors are positive lymph nodes, parametrial invasion, or positive surgical margins.^2^ According to Sedlis criteria, the criteria for intermediate‐risk tumors was as following: the presence of LVSI plus deep (outer third) cervical stromal invasion and tumor of any size; the presence of LVSI plus middle (one‐third) stromal invasion and tumor size ≥2 cm; the presence of LVSI plus superficial (inner third) stromal invasion and tumor size ≥5 cm; or no LVSI but the deep or middle cervical stromal invasion and tumor size ≥4 cm.^8^ Low‐risk was defined as the absence of high‐risk factors and intermediate‐risk factors. About 626 patients were confirmed as low‐risk squamous cervical cancer. Fifteen patients who received any neoadjuvant treatment before surgery, 198 patients lost to follow‐up, and 46 patients without differentiation details were excluded. Finally, 367 low‐risk squamous cervical cancer patients with full following up details were included in this study.

Among these 367 patients, the standardized manner of differentiation was recorded according to the following criteria: 1) in the highly differentiated (G1) group, tumor cell nests were composed of keratinocyte‐like cells with easily visible keratinization features (layered or cytoplasmic keratin); 2) in poorly differentiated (G3) group, squamous morphology was only noticeable in a small area of the tumor; 3) in the moderately (G2) differentiated group, tumors showed an intermediate degree of squamous differentiation that was between the highly and poorly differentiated ones,^9^ (Supplemental Figure S1). Recent studies reported that two‐tiered conventional grading had a greater impact on prognosis than the three‐tiered conventional grading system.^9^, ^10^ Accordingly, we merged G1 and G2 groups into one group. The study was approved by the institutional human ethics committee of the First Affiliated Hospital of Wenzhou Medical University. All patients agreed and obtained written informed consent.

### 2.2 Treatment

All included patients received radical hysterectomy and pelvic lymphadenectomy. After pathological examination, low‐risk squamous cervical cancer was further classified into two groups according to differentiation (G1/G2, G3). Patients with G1/G2 were advocated routine follow‐up. None of these patients received adjuvant therapy. Concerning poorly differentiated tumors (G3), there are currently different views in our center. Doctors will choose different treatment strategies based on their own experience: following up or receiving chemotherapy. Thus, while 114 patients received no adjuvant therapy after surgery (nonchemotherapy group), 47 patients received chemotherapy (chemotherapy group). Patients in the chemotherapy group, received 5‐Fluorouracil plus cisplatin treatment (2008–2009) or paclitaxel‐cisplatin treatment (2010–2018) 10–15 days after surgery. Five patients received four cycles of 5‐Fluorouracil (4000 mg/m^2^) and cisplatin (75 mg/m^2^) once every 4 weeks. Forty‐two patients received four cycles of paclitaxel (135 mg/m^2^) and cisplatin (70 mg/m^2^) once every 3 weeks. The toxicity of chemotherapy was manageable. No patients died from side effects.

### 2.3 Follow‐up

Follow‐up intervals were scheduled as follows: every 3–6 months for the first 2 years, every 6–12 months for the next 3 years, and yearly thereafter based on NCCN Guidelines.^11^ Follow‐up evaluation included physical examination, imaging, Pap smears, and complete blood counts. Besides, an assessment of symptoms or treatment complications was performed when indicated. Recurrence and overall survival were calculated from the date of surgery to the date of recurrence/death or the date of the last follow‐up visit. We compared recurrence‐free survival (RFS) and overall survival (OS) as primary endpoints between the chemotherapy group and the nonchemotherapy group. The follow‐up phase lasted until November 2019.

### 2.4 Statistical analysis

Fisher's exact or χ^2^ test analyzed categorical proportions between the nonchemotherapy groups and chemotherapy groups, respectively. All statistical tests were two‐sided, and *p* < 0.05 was considered statistically significant. Survival analysis was analyzed with Kaplan–Meier method. Difference in survival were analyzed with Log‐rank tests among different groups. SPSS version 20.0.0 software was used for statistical analysis.

## 3 RESULTS

### 3.1 Survival outcomes according to the differentiation among low‐risk patients

This retrospective study included 367 patients, two hundred and six patients (56.1%) were G1/G2, and 161 (43.9%) were G3. None of the patients in the G1 and G2 groups received adjuvant treatment after radical hysterectomy. About 47 patients received chemotherapy, the other 114 patients received no adjuvant therapy after radical hysterectomy in the G3 group. Patient characteristics of the G1/G2 group and G3 group are shown in Table 1. There showed no significantly different characteristics between the two groups.

**TABLE 1:** Clinical characteristics of squamous cervical cancer patients with low‐risk factors

Parameter	G1/G2 N = 206	G3 N = 161	*p*‐Value
Age (y)	50 (44, 58)	52 (44, 61)	0.523
FIGO stage			0.771
I	155	119	
II	51	42	
Tumor size			0.394
<2 cm	143	105	
2–4 cm	63	56	
Type of surgery			0.456
Open	179	144	
Laparoscopic	27	17	
Recurrence			0.014*
Yes	3	10	
No	203	151	
Death			0.050
Yes	3	8	
No	203	153	

Abbreviation: N, number of patients.

* *p *< 0.05.

**FIGURE 1:**
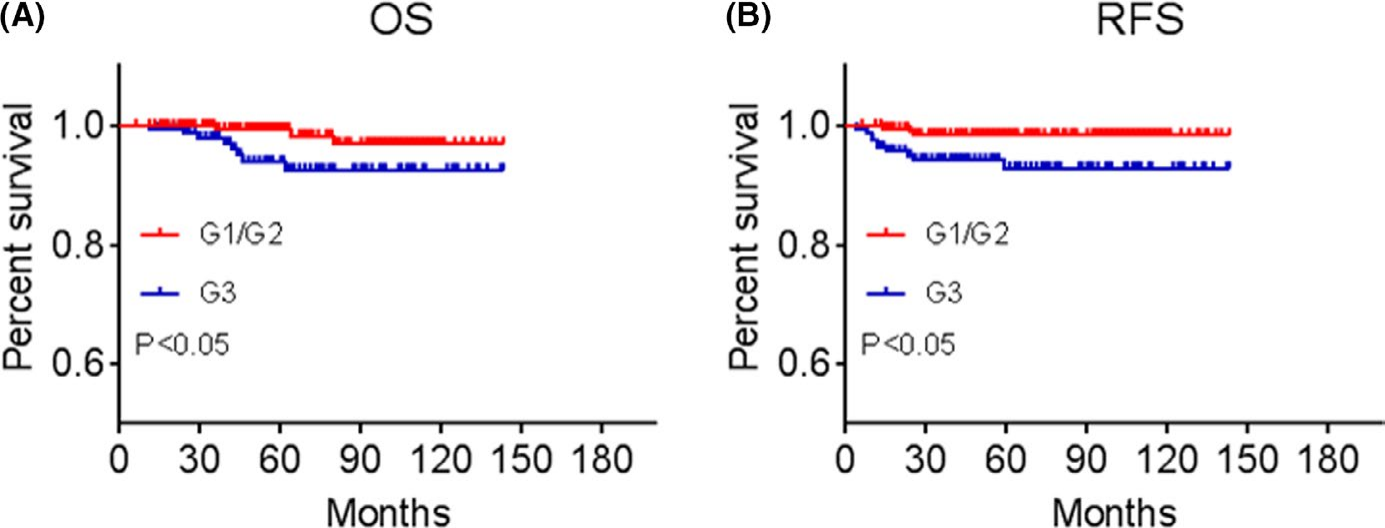
Kaplan–Meier plot for (A) overall survival (OS) and (B) recurrence‐free survival (RFS) in G1/G2 groups versus G3 group among patients with low‐risk recurrence of squamous cervical cancer

The median follow‐up duration for all patients was 56 months (interquartile range 34–94 months). Eight death was occurred in the G3 group, of which six were disease‐specific death. In the G1/G2 group, three death occurred, only one patient suffered disease‐specific death. The 5‐year overall survival rates of G1/G2 group and G3 group were estimated to be 99.4% and 93.9%, respectively. AS shown in Figure 1A, patients with G3 displayed a significantly poor survival (hazard ratio 3.65, 95%CI 1.14–12.53, *p* = 0.035) than those with G1/G2 (Figure 1A).

Three patients were observed recurrence in the G1/G2 group (206 patients) and 10 (6.2%) patients in the G3 group (161 patients). The 5‐year recurrence‐free survival rate in the G1/G2 group and G3 group was estimated to be 98.4% and 92.9%, respectively. AS shown in Figure 1B, the patients with G3 displayed a significantly higher recurrence rate (hazard ratio = 4.38, 95%CI = 1.46–13.13, *p* = 0.014) than the patients with G1/G2 (Figure 1B).

### 3.2 Survival outcomes according to adjuvant chemotherapy among low‐risk patients with poor differentiation

There were 161 squamous cervical cancers pathologically confirmed as low‐risk and poor differentiation (G3). In which, 47 (29.2%) of patients received chemotherapy after radical hysterectomy, and 114 (70.8%) did not receive any treatment after surgery. The characteristics of the two treatment groups are summarized in Table 2. Neutropenia was observed in 9 (19.1%) patients in the chemotherapy group (47 patients), while other chemotherapy side effect like peripheral neuropathy was not occurred. There were no significant differences in other characteristics between the groups.

**TABLE 2:** Clinical characteristics of low‐risk squamous cervical cancer patients with poor differentiation

Parameter	Non Chemotherapy N = 114	Chemotherapy N = 47	*p*‐Value
Age (y)	53 (45, 61)	51 (43, 58)	0.1661
BMI	21.55 (18.1, 33.2)	22.7 (17.7, 30.9)	0.081
ECOG grade			0.11
0	78	38	
1	36	9	
SCC value	1 (0.02, 7.7)	1 (0.4, 21.2)	0.122
Triglyceride	1.68 (0.44, 9.04)	1.48 (0.35, 9.6)	0.217
Cholesterol	4.94 (2.9, 8.94)	4.68 (0.55, 9.3)	0.021
FIGO stage			0.619
I	83	36	
II	31	11	
Tumor size			0.899
<2 cm	74	31	
2–4 cm	40	16	
Type of surgery			0.250
Open	104	40	
Laparoscopic	10	7	
Recurrence			0.082
Yes	10	0	
No	104	47	
Death			0.143
Yes	8	0	
No	106	47	
Chemotherapy side effect (Neutropenia)			0.000*
Yes	0	9	
No	114	38	

Abbreviation: N, number of patients.

*p < 0.01.

**FIGURE 2:**
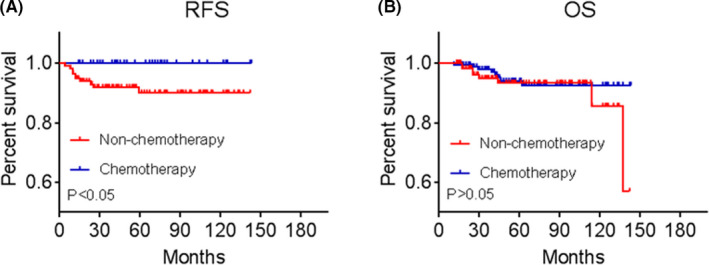
Kaplan–Meier plot for (A) recurrence‐free survival (RFS) and (B) overall survival (OS) in nonchemotherapy group versus chemotherapy group among poor‐differentiated patients with low‐risk squamous cervical cancer

The median follow‐up duration for all these 161 patients was 50 months (interquartile range 30.4–79.9 months). About 10 of 161 (6.2%) low‐risk cervical squamous cancer patients with poor differentiation suffered a recurrence. Intriguingly, all these 10 RFS events occurred in the nonchemotherapy group, while none recurrence was occurred in the chemotherapy group, the 5‐year RFS rates of two groups were 90.0% and 100%, correspondingly. Kaplan–Meier analysis revealed that chemotherapy group was significantly associated with a better RFS than those in the nonchemotherapy group (hazard ratio = 0.24, 95 CI = 0.06–0.93, *p* < 0.05) (Figure 2A).

Of these 161 patients, only eight patients died in the nonchemotherapy group at the time of the last analysis, and no death occurred in the chemotherapy group, with 5‐year OS rates of 94% and 100%, correspondingly. Kaplan–Meier analysis showed no significant difference in overall survival between the chemotherapy group and the nonchemotherapy group (*p *=* *0.408) (Figure 2B). The median duration from recurrence to death was 13 months (range, 3–21 months).

### 3.3 Pattern of disease recurrence

In the G1/G2 group, one case experienced vaginal stamp recurrence, one case was pelvic metastasis, and one was lung metastasis. In G3 group, mainly in nonchemotherapy group, recurrence of disease was detected in the vaginal vault (n = 2), pelvic side wall (n = 5), and pelvic side wall with rectum (n = 1). One patient had synchronous relapse in her lung, hilar lymph nodes, and bone. One patient recurred in the lung. Thus, the rate of vaginal recurrence was 23.1% (3/13), pelvic side wall recurrence was 53.8% (7/13), and distant relapse was 23.1% (3/13). The median time of recurrence was 12 months. Only one patient with lung metastasis recurred after surgery in 59 months. The other seven patients had recurrence within 2 years after surgery.

## 4 DISCUSSION

Our current study evaluated, for the first time, the oncological results of adjuvant chemotherapy following radical hysterectomy among low‐risk squamous cervical cancer with poor differentiation, and found that postoperative chemotherapy may significantly improve the survival of low‐risk squamous cervical cancer patients with poor differentiation.

Numerous clinical trials have explored risk factors for recurrence and survival among cervical cancer patients. Pelvic node status, surgical margins, and parametrial involvement were identified as high‐risk factors,^2^ and tumor size, depth of stromal invasion, and lymphovascular space involvement were identified as intermediate‐risk factors.^8^ Some studies have also demonstrated that differentiation significantly affects progression‐free survival (PFS) and survival, especially for patients without high‐risk and intermediate‐risk factor. In early 1991, Smiley et al. identified the degree of differentiation as the only histopathologic factor correlated with recurrence among patents with stage IB squamous cervical cancer with low risk for recurrence.^5^ Similar results were reported by Delgado, G.^4^ Recently, Matsuo et al. analyzed 4,045 low‐risk squamous cervical cancer (stage I, without lymph node metastasis, tumor size ≤4 cm) through the surveillance, epidemiology, and end result program, and reported that the cause‐specific survival increased to 4.48 times among patients with poor differentiation as compared to those with well‐differentiation.^6^ In agreement with previous data, our results showed that among low‐risk squamous cervical cancer, patients with G3 differentiation showed worse RFS as well as OS than those with G1/G2 differentiation. One of the probable reasons for these findings is that keratin patterns are an integral part of clustering squamous cervical cancer and are related to survival.^12^

Chemotherapy, as an adjuvant therapy, has been widely used in terms of cervical cancer. Cisplatin‐based chemotherapy had become the standard treatment for patients with recurrent or metastatic cervical cancer, in which cisplatin combined with paclitaxel was the most effective chemotherapy regimen for recurrent, or metastatic cervical squamous cell carcinoma to date.^13^, ^14^ Besides, a recent meta‐analysis showed that adding platinum‐based chemotherapy to adjuvant radiotherapy may improve survival rate and risk factors for recurrence in women with early‐stage cervical cancer (IA2–IIA).^15^ Adding fluorouracil and cisplatin chemotherapy to external‐beam and intracavitary radiotherapy can significantly improve the survival rate of women with locally advanced cervical cancer.^16^ Furthermore, neoadjuvant chemotherapy was a reasonable option to delay delivery for cervical cancer patients during pregnancy.^17^ What is more, although neoadjuvant chemotherapy followed by radical surgery did not show superior than concurrent chemoradiation in locally advanced cervical cancer,^18^ the response rate presented 80%.^19^ Collectively, these results support the notion that cisplatin‐based chemotherapy showed efficiency either in preventing recurrence or in controlling disease progression. Recently, we detected that postoperative chemotherapy may significantly improve the survival of patients with these diseases. The addition of chemotherapy greatly reduced local and distant recurrences of cervical cancer, improving the recurrence‐free survival rates. The difference in RFS between chemotherapy compared with nonchemotherapy deserves consideration in treatment planning and future clinical trial design. However, adjuvant treatment is associated with adverse toxicities, such as rectosigmoid with proctitis, tenesmus, diarrhea, fistula, stenosis, and ulceration. Toxicity within bladder, local dermal toxicity, gynecological toxicity, acute hematological, and gastrointestinal toxicity have also been reported in the patients treated with adjuvant therapy.^20^

Although chemotherapy decreased the risk of recurrence, adjuvant therapy showed no effect on overall survival. Low‐risk squamous cervical cancer patients likely have well overall survival and low mortality. Besides, the majority of recurrences (8 of 10 recurrences, 80%) were local. Patients with local metastasis usually have a lower risk of death compared with those lymphatic metastasis.^21^, ^22^ Also, the relatively short follow‐up duration is also a cause of no difference in survival time.

Several studies have explored the necessary of local therapy among cervical cancer. Hee‐Sug et al. reported that postoperative concurrent chemoradiotherapy for stages IA2–IIA cervical cancer patients with high‐risk factors had comparable 5‐year overall (96.7% vs. 97.7%) and progression‐free survival rates (88.7% vs. 95.4%) compared with control group who had no high‐risk factors and received no adjuvant therapy after surgery.^23^ Liu et al. found that adjuvant chemoradiotherapy showed better 5‐year survival rate (90%) than radiotherapy alone (76%) for patients with high‐risk IB and IIA stage cervical cancer who underwent surgery.^24^ Maureen et al. also showed that stage IB/IIA cervical cancer patients treated with a platinum‐based regimen of chemotherapy and radiotherapy had better outcomes (no recurrences with 37.1 mean follow‐up time) than patients received radiation alone (two had recurrences).^25^ It is worth noting that, in G3 patients/ nonchemotherapy arm, seven out of eight recurrences were local and within two years from surgery, only one patient had distant failure that too after nearly 5 years, suggesting local therapy may be importance in case an adjuvant is felt necessary in the G3 tumor. In the future, more attention should be paid to local radiotherapy as adjuvant therapy in the G3 tumor.

Several limitations to the current study have to be mentioned. This research is a retrospective study, the adjuvant therapy ways were not random allocated, errors should be considered. Second, the sample size is small, especially in the chemotherapy group (n = 47). Besides, we did not perform cost effectiveness analysis, since low‐risk squamous cervical cancer patients showed a good survival, and almost half of patients had poorly differentiated tumors. Finally, the follow‐up is not long enough. Although, there is no sufficient evidence to recommend that low‐risk squamous cervical cancer patients with poor differentiation confined to adjuvant chemotherapy, the difference in RFS between chemotherapy compared with nonchemotherapy deserves consideration in treatment planning and in the design of future clinical trial.

In conclusion, patients with poor differentiation were correlated with poor clinical outcomes compared with those with good differentiation. Cisplatin‐based adjuvant chemotherapy resulted in a significantly decreased RFS rate compared with nonchemotherapy following radical surgery among low‐risk squamous cervical cancer patients with poor differentiation. Future randomized controlled trials with large sample size are necessary to confirm these results.

## CONFLICTS OF INTEREST

The authors report no potential conflicts of interest.

## AUTHOR CONTRIBUTIONS

Conception and design: Haiyan Zhu and Hongqin Zhao; Collection and assembly of data: Qi Jiang, Xinxin Xu, and Anyang Li; Data analysis and interpretation: Haiyan Zhu and Hui Luo; Manuscript preparation: Hongxia Yao and Hui Luo.

## Supporting information

Fig S1

## Data Availability

All data are available upon request.
