# Supplementary figures and images for: Prognostic significance of poorly differentiated histology and impact of adjuvant chemotherapy in early squamous cell carcinoma of cervix uteri
Source: Cancer Med. 2021 Mar 18;10(8):2611–7. doi: 10.1002/cam4.3780 (PMC8026924; doi:10.1002/cam4.3780)

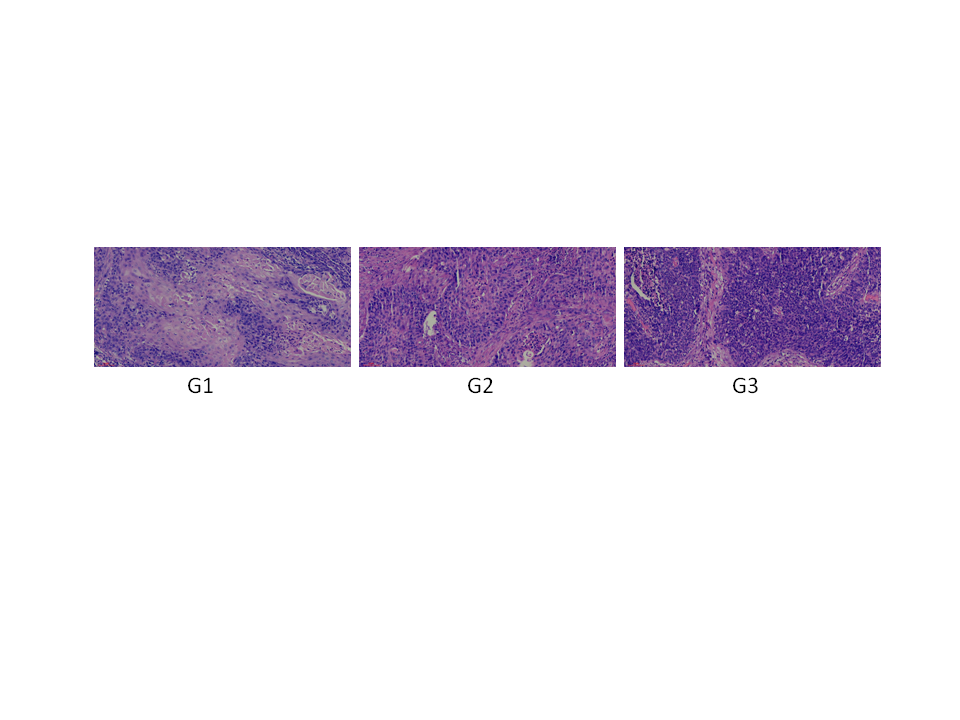

Supplement: Supplementary file 1 — Fig S1 [file CAM4-10-2611-s001.tif]
